# Observations on Insanity, and Its Treatment in Private Practice

**Published:** 1846-10

**Authors:** John Evans

**Affiliations:** Professor of Obs., &c., in “Rush Medical College,” Chicago


ILLINOIS AND INDIANA

MEDICAL AND SURGICAL JOURNAL.

Vol. I. NEW SERIES.—OCTOBER, 1846. No. 4.

### PART I.—ORIGINAL COMMUNICATIONS.

### ARTICLE I.

*Observations on Insanity, and its Treatment in Private Practice.* By JOHN EVANS, M. D., Professor of Obs., &c., in "Rush Medical College," Chicago.

The subject of Insanity, from the number of institutions exclusively devoted to its treatment, and of those engaged in its investigation, has almost become a separate branch of medical science. This tends to two opposite results; the one of great utility, and the other prejudicial to the best interests of the insane. From the devotion of a large body of able physicians to the investigation of the pathology and treatment of the disease, important discoveries have been made, and almost a complete reformation in the treatment of the disease, and in public opinion has been effected. But, on the other hand, from the fact of these institutions and physicians being exclusively engaged on the subject, the great body of the profession pay much less attention to it, and are disposed to leave it entirely in the hands of hospital physicians.

While all will admit that a well regulated hospital for the insane is the proper place for their treatment, it should be remembered that many cases may be well treated and speedily restored by proper management in private practice. The insane often suffer from a want of attention to the subject of insanity, by the great body of the profession. It will not unfrequently be found that this state of things has prevented the necessary remedies from being applied, until the patient has become incurable, or has caused him at great expense and loss of time in treatment, to be sent to some remote hospital, where, had a timely application of those means at the command of the general practitioner been resorted to, he would have been speedily restored. It is in view of this consideration, that I propose calling the attention of the profession in the west to the subject at this time, regarding it of the greatest moment here, where we are almost entirely unprovided with hospitals for the insane, and where it is entirely beyond the ability of large numbers of this unfortunate class of patients, to obtain admission into such institutions.

I shall briefly consider some of the causes, symptoms, especially of the incipient stage, and pathology, and then speak more particularly of the treatment proper to be adopted in private or general practice.

*Causes of Insanity.*—Amongst the causes that have been enumerated, may be found almost every unpleasant mental condition and physical irregularity. They may be divided into two classes: *the physical* and *the moral* or *mental.*

Although it is difficult to classify, yet, this will be found to present as clear a line as can be drawn. The reciprocity of the two classes of causes, may in many cases be close, as there must be physical derangement before insanity can be established, and the influence that may be exerted by moral causes, will be often productive of a train of disease before the insanity is developed. But, notwithstanding these difficulties, this is the only classification of the causes, that they will in my mind admit of, without going into long hair-splitting definitions, that would more embarrass the subject than such classifications would elucidate it.

*The physical causes,* include all those that make the primary impression from which the insanity results upon the physical man; as, when disease of the stomach has produced insanity, without any direct or powerful mental impression. There are cases which it would be difficult to determine, to which class they belong; but not more than of those for whose insanity no cause at all can be assigned,—and others that seem to be produced by both physical and moral causes.

Most of the diseases of the system have been known more or less, directly, to produce insanity; but there is a great difference in this respect, for while some but seldom have been known to have this effect, others frequently produce it.

From what has been said it is plain that an almost countless variety of physical influences may be properly considered under this head.

Amongst them are climate, varieties in the species, seasons, intemperance, modes of life, errors in education, age, temperament, injuries of the head, sudden arrest of perspiration, healing of issues, metalic influences, disorders of the menses, puerperal influences, the critical period, worms in the intestines, fevers, dyspepsia, apoplexy, epilepsy, hereditary predisposition, indulgence in vicious habits, as masturbation, prostitution, &c.

*Climate.*—There is perhaps less direct influence in the production of insanity in the climate, than that that is exerted in the formation of an energetic and vigorous character, and the reverse. We find in those countries where the climate is such as to produce a languid and dull condition of the moral and physical man, as in tropical climates, insanity is less frequent; while in the temperate latitudes we find the disease much the most common. Although insanity is said to prevail to a great extent in the British East Indies, it must be recollected that the inhabitants are northern in their origin, education and habits. The following statistical table, derived from various sources, is as nearly correct as the information on the subject will enable me to make it.

TABLE No. 1.

Showing the proportion of Insanity to the population in different countries and latitudes.

| | | | | | | | | | |
|---|---|---|---|---|---|---|---|---|---|
| Norway, bet. | the 58th | and | 80th | Deg. of N. Lat. | has insane, | 1 | to every | 551 |
| Scotland, | " | 55th | " | 59th | " | " | 1 | " | 563 |
| England, | " | 50th | " | 55th | " | " | 1 | " | 793 |
| U. States,* | " | 30th | " | 48th | " | " | 1 | " | 985 |
| Netherlands, | " | 50th | " | 54th | " | " | 1 | " | 1050 |
| France, | " | 43d | " | 50th | " | " | 1 | " | 1000 |
| Italy, | " | 40th | " | 46th | " | " | 1 | " | 4879 |
| Spain, | " | 36th | " | 44th | " | " | 1 | " | 7181 |

* This is taken from the census of 1840, and includes the insane and idiotic.

While this table shows that in the higher latitudes there is a greater proportion of insanity, it must be remembered that there are many other circumstances, that have great influence in modifying the results.

Even in the United States, there are both moral and physical causes influencing the results independent of climate, but that it has an influence will be apparent from a table, made out from the census of 1840, in another part of this article.

*Varieties of the race.*—Considering the human family as divided into five varieties, it will be reasonable to expect that they will, as they are varied in character and habits, be different in their liability to insanity. While we have in reference to some of the varieties but little positive information, we are warrantable in the conclusion that the Caucasian is by far the most obnoxious to the disease. Of the Mongolian, we can only give the reports of travellers, who say that insanity is very rare in China. Of the aboriginal American we also have but little information. Baron Von Humboldt found but few cases among them, and from sources of information of a more recent date, we have nothing that in the least goes to controvert the opinion drawn from his observations. Of the Malay or South Sea Islander, we have no positive information, except from Capt. Wilkes. In a letter to Dr. Brigham, he says, "During the whole of my intercourse among the natives of the South Sea, I met with no deranged person, and I am satisfied that insanity is a disease incidental alone to civilized life. I am confident that had any instances of mental derangement among the natives occurred, it would have been observed by us."

By an estimate of the number of insane among the blacks, of course including mulattoes and all other degrees of the admixture of the white and black races, we find that there is in the United States, as reported in the census of 1840, one colored person insane or idiotic in every 982.

By a reference to table number 1, it will be seen that of all the inhabitants of the United States there are insane or idiotic, one in every 985, and of course the result would be but little varied if we would from the same data make out the per cent. of insane and idiotic among the white inhabitants. From all that can be learned, we are justifiable in the conclusion that the African in his native country, is almost exempt from insanity.

*Seasons.*—The only means we have of determining the influence of the seasons in causing insanity, is deduced from admissions at different seasons into the different institutions for their treatment; and it must be remembered that other circumstances have much influence in determining these results. Patients are not generally admitted at any particular period after the attack. The statistics show more admissions during the summer months. But heat being favorable to excitement, and travelling being better during this season, may have more influence in producing the result than the periods of attack.

*Intemperance.*—This curse of our race, as in almost all other diseases, so in insanity, is found to be one of the most fruitful causes. And amongst the physical it is by far the most prolific of all others. So far as statistical information has been obtained, this is not only true of this country, but also of all others.

The immediate intoxicating effects of *ardent spirits*, are such as to lead us at once to infer that their continued influence would soon derange the mind. There is a species of derangement peculiar to drunkenness, which is not included by the definition of insanity as here treated, called *mania a potu*, of which I do not propose to speak. The remarks I make refer to insanity, strictly so called.

Intemperance in the use of *opium*, too, might be expected a priori to produce insanity. The powerful impressions made on the brain and nervous system by this drug, are such that when long continued they often result in derangement of the mind.

Intemperance in the use of *tobacco*, which also has a powerful influence over the nervous system, might be expected often to result in insanity. And in reference to it Dr. Woodward says, "that tobacco certainly produces insanity I am not able positively to observe; but that it produces a predisposition to it I am fully confident."

*Modes of life.*—As there is a marked influence exerted on man by his manner of living, both physically and mentally, some modes seem more than others favorable for the development of insanity.

Sedentary habits are destructive of that physical energy necessary to health, and are by most authors regarded as a cause of insanity. Keeping irregular hours, too, might with great propriety be noticed in this connexion; for all agree that the habit has a tendency to undermine the health, and disturb the equilibrium of the mind. In fact all excesses may be regarded as tending to produce insanity where there is a predisposition to it. Luxurious living is favorable to it, as well as a meagre diet. In proof of this, we find in Europe the highest and lowest classes, more particularly, are liable to the disease.

*Errors in Education.*—Physical education is more necessary to the well being of the individual, than mental; for without it, he cannot enjoy health of body or vigor of intellect. Parents too often overlook this valuable truth, and hoping to make intellectual prodigies of their children, tax the mind to the utmost, while the body and limbs are left without that exercise which is so necessary to their development. And while they often succeed in inducing a precocity of mind, they lay the foundation for early disease and death; or institute an irritation in the brain to result in insanity or imbecility. The part of the system most exercised, during development especially, grows much faster than others; and by early taxing the brain, it is too rapidly developed, and disease, either in the brain or some part of the enfeebled and imperfectly developed system, is the consequence. How few of the many remarkably intelligent children, ever arrive at the age of maturity. And of those that do, how very few, realize the bright hopes predicated upon their precocity. Children, and especially those that give evidence of premature brilliancy of intellect, should not be put to study until they are so far developed, that the physical system can endure the fatigues of mental applications. Until the age of seven or eight years, the brain is exceedingly delicate in its texture, and of but little more than a fluid consistence. Is it then strange that the general practice of modern times of forming infant schools, and by other means urging the child forward in education with hot-house rapidity,—placing it under all possible excitements to induce it to apply its mind to study, should lay the foundation for disease, insanity, imbecility, or idiocy?

Again as the integrity of the mind depends upon the health and regular development of the instrument by which it acts, it is necessary that in education care should be had that the different faculties or powers, or the organs through which they act, shall be properly and regularly exercised and developed. Otherwise we cannot expect a well ballanced mind, without which there will be a predisposition to insanity. A child that is educated to a frequent indulgence of anger, while the controuling sentiments are suffered to lie dormant, will be strongly predisposed to insanity by this great error in its education. And so with other passions and faculties—when one is inordinately developed and others neglected, it will constitute a strong predisposition to derangement of the mind.

*Age.*—The period of youth is almost entirely exempt from insanity. I saw a little boy in the N. Y. State Lunatic Asylum, last spring, about twelve years of age, who was quite deranged. But such cases are exceptions to the general rule. Dr. Earle says "such cases are extremely rare." Dr. Rush mentions four cases of the kind, which came within his knowledge, and in St. Luke's Hospital, England, there was an insane child of but *two years* of age. Old age is pretty well exempted too, except from senile insanity, which does not come under this head. More persons are attacked between the ages of twenty-five and forty, than at any other period in life. It is then that man acts most, and is most subjected to the reverses of fortune and vicisitudes of life, that exercise such influences in producing this disease.

*Temperament.*—The sanguine and nervous temperaments are most favorable for the development of diseases of the mind. The first on account of the impetuosity of action in the system generally peculiar to it, and the other because of its excitability. Many of our best writers say that the bilious or choloric are particularly subject to mania. There have been some curious statistics collected in reference to the color of hair, eyes, and the complexion of the insane, but as nothing definite has been made out from them, I will not trouble my readers with their perusal.

That *injuries of the head* should produce insanity, is no more strange, if they affect the brain, than that those of the eye should impair sight, or of the ear hearing. And although the brain may sustain injuries of considerable extent, without a derangement of the mind, it is stated by some of the highest authorities, that no case of the kind has occurred, where corresponding portions of the hemispheres were injured or diseased at the same time. If we admit the brain to be the organ of the mind, which few if any doubt, this is what we would expect, reasoning from analogy.

*A sudden arrest of perspiration and the healing of issues* have frequently been followed by a derangement of the mind.

*Metalic influences.*—Esquirol, after observing that persons exposed to the heat of the sun, and the fumes of charcoal, are subject to insanity, says: "Those who are obliged to work in the midst of metalic oxyds, cooks and miners, are liable to the same attacks.

"The vapour of lead, produces in Scotland a species of insanity, in which the maniacs lacerate themselves at every opportunity, and which the Scotch peasants call Mill-reeck.

"The miners of Peru and Mexico, are subject to a peculiar form of insanity."

*Disorders of the menses, puerperal influences and the critical period.*—From the powerful influence exerted by the uterus and ovaries over the nervous system of the female, are prolific causes of insanity. Esquirol says, nearly one-twelfth of the women at the Salpêtrière, became insane after confinement.

*Worms.*—The entozoa, in consequence of the irritation they produce, and the intimate sympathy between the brain and abdominal visceræs, are often found holding a place amongst the causes of insanity.

*Fevers.*—That insanity should frequently result from fevers is reasonable, in consequence of the general morbid influence exerted by them; and the liability, not only of all the organs of the system to suffer lesions from them, but particularly in consequence of the effects of these forms of disease upon the brain itself.

I was consulted about eighteen months ago, in reference to the case of a young man, of active mind and devoted business habits, he being an extensive grazer in Illinois; who in consequence of a violent attack of remittent fever, and great anxiety in reference to his business, had become insane.

By the use of the proper remedies for the fever, prescribed by his physicians, his mind had been temporarily restored; but by too early application to his business, and a relapse of the bilious derangement of the system, his insanity returned upon him. He was early taken to an Eastern Lunatic Asylum, and was speedily restored to health.

*Dyspepsia.*—The intimate sympathy through the pneumogastric nerve, existing between the stomach and brain, and their dependence through digestion and assimilation, and the influence they exert over the circulation, would lead us to the conclusion, that disease of the stomach would exert a morbid influence over the brain, and be liable to produce insanity.

*Apoplexy.*—The violence done to the brain by this disease, is very liable to result in insanity.

*Epilepsy.*—This is a very common cause of insanity of the most deplorable character. The insanity assumes peculiar forms; generally being most violent after the attacks of epilepsy, and either moderating or entirely subsiding in a longer or shorter time after the convulsions. This is one of the most hopelessly incurable forms of the disease. When once established, seldom giving way to any form of treatment.

*Hereditary predisposition, or conformation.*—That there is a liability in the progeny of parents affected with insanity, to the disease, is proved by a long train of observations, which have been handed down to us by the highest authorities.

In this cause of, or predisposition to insanity, we find many curious facts. Esquirol says the taint in the system is oftener transmitted from the mother than the father.

It has often been remarked that those hereditarily predisposed to insanity of the same family, are often attacked with it at the same period in life. Burton says that children of aged parents are predisposed to melancholy. "Children who are born before their parents become insane," says Esquirol, "are less liable to mental alienation than those whose birth takes place afterward." The same is true of those who are born of parents who are insane only on the paternal or maternal side, compared with those both of whose parents are insane."

There is generally great similarity in the symptoms in those who become insane of the same family.

In addition to the hereditary influence predisposing to insanity, there is an acquired peculiarity of constitution, which favors the development of this disease. Pritchard thinks this obtains in all cases of insanity where there is no hereditary taint.

*Vicious habits, Masturbation, Prostitution, &c.*—By looking over the reports of the American institutions for the insane, I was surprised at finding such a large number of cases reported as being caused by masturbation. This cause is almost entirely confined to males; a few females only, being reported under this head. Esquirol says it is because women are more reserved than men. There is, I apprehend, great uncertainty in reference to the amount of insanity caused by this *secret* vice, as many may become addicted to it after insanity supervenes. Those addicted to the vice, whether causing the insanity or not, are in consequence of it difficult of cure.

From the diseases prostitution is liable and almost certain to induce, not only in the organs of generation but of the general system, it is wòrthy of a place amongst the physical causes; yet there is no doubt of the powerful moral or mental influence excited by the degradation which is a necessary attendant upon it.

Esquirol says: "One twentieth of the alienated admitted at the Salpêtrière, have been prostitutes. The proportion is much larger in Paris than in any other place.

### MORAL CAUSES.

Although as before remarked, physical disease or malformation, is necessary to insanity, it is found that much the larger number of cases can be traced, as an immediate cause, to moral or mental influences. These seem most frequently to coöperate with physical disease, (which is the only actual disease,) in the production of mental derangement.

Those conditions and influences that, by producing an excitement of the mind, predispose to, and cause insanity, like the physical derangements of the system, are so numerous that it would be tedious to enumerate them. A few of the most common of these may be considered under the heads of—the moral condition, the social and intellectual condition, the political and religious condition, war, the civil state, disappointed love, domestic troubles, grief, jealousy, anger, fright, fanaticism, reverses of fortune, disappointed ambition, mortified pride, excessive study, &c.

*The moral condition* of a people has much to do in the production and prevention of insanity. It is found that in those countries where the people are addicted to vicious habits,—other things being equally favorable—they are much more subject to mental alienation, than where a more wholesome state of moral feeling, and virtuous living are found. And why should we expect it to be otherwise? Does not vice lead to the excesses that derange the general health? And is it not the foundation and source of all misery? Then, as a people are vicious, will they be exposed to more physical disease, and more of the evil inclinations and propensities of the heart, which produce sorrow and woe, will influence their lives, and in the train of miseries will often be found insanity. It may be that from this fact, originated the idea that the insane are "possessed"—that they are under the influence of the "evil one," for when it originates in vice, it is in a certain sense true.

As *the social and intellectual* condition of man influences to a great extent his mind, it has much to do in predisposing to and exciting insanity. In those countries where there is little mental culture—where the inhabitants are ignorant, and consequently can have but little to excite the mind, insanity is rare. The untutored savage is seldom insane. He is free from the moral and intellectual commotions that so frequently shake civilized society to its centre. His troubles are principally physical, and as a compensation for the loss of those higher and more exalted enjoyments flowing from the cultivation of the mind, he is more exempt from its diseases.

By a comparison of the proportion of insanity amongst the colored population of the United States, that are in slavery, and those enjoying freedom, as shown by the census of 1840, I find that amongst the blacks in the slave states, there is only 1 insane in every 1557; while in the free states there is 1 insane in every 163. This is the result after correcting the error in the report of Massachusetts, and in a remarkable manner denotes not only the influence of the social condition in producing insanity, but also shows that ignorance affords in a degree exemption from it.

The slave is to a great extent free from the cares and mental anxieties of a life of freedom. As a general rule, in the United States at least, he is provided with wholesome diet in abundance, without the necessity of looking after it; so that the disease and distress that obtain in the lower classes in Europe, where insanity is rife, are comparatively unknown amongst the slaves of our country. There are, however, causes for insanity here of no ordinary character, which, if obtaining amongst a people of intelligence and cultivated feeling, would fill the hospitals, poor-houses, and jails, with their victims. I mean the practice of severing families assunder—breaking the strongest ties of affection, by the rude hand of force and other abuses incident to slavery.

The following table shows the proportion of insanity amongst the negroes of the United States. Although from some gross errors that have been detected in the census of 1840, from the report of which this has been taken, some are disposed to cast the whole aside as untrue; I think sufficient reliance may be placed on it to lead us to an approximation of the truth in general results. In the calculation I have omitted fractions.

TABLE, No. 2.

Showing the colored population of each State and Territory in the United States, the number of insane and idiotic among them, and the ratio of sanity to insanity.

OF THE FREE STATES.

| | Whole colored population. | Insane and Idiotic. | Propor'n of Insane & Idiotic to sane. |
|---|---|---|---|
| Maine, | 1,355 | 94 | 1 to every 14 |
| New Hampshire, | 538 | 19 | 1 " " 28 |
| Massachusetts, | 8,689 | 67 | 1 " " 129 |
| Rhode Island, | 3,243 | 13 | 1 " " 249 |
| Connecticut, | 8,159 | 44 | 1 " " 183 |
| Vermont, | 730 | 13 | 1 " " 56 |
| New York, | 50,031 | 194 | 1 " " 257 |
| New Jersey, | 21,718 | 73 | 1 " " 298 |
| Pennsylvania, | 47,918 | 187 | 1 " " 256 |
| Ohio, | 17,345 | 165 | 1 " " 105 |
| Indiana, | 7,168 | 75 | 1 " " 96 |
| Illinois, | 3,929 | 79 | 1 " " 49 |
| Michigan, | 707 | 26 | 1 " " 27 |
| Wiskonsin, | 196 | 3 | 1 " " 65 |
| Iowa, | 188 | 4 | 1 " " 47 |
| Totals, | 171,914 | 1,056 | |

Making 1 to every 163 in the Free States.

OF THE SLAVE STATES.

| | | | |
|---|---|---|---|
| Maryland, | 151,515 | 141 | 1 to every 1,074 |
| Virginia, | 498,829 | 381 | 1 " " 1,309 |
| Delaware, | 19,524 | 28 | 1 " " 697 |
| N. Carolina, | 268,549 | 221 | 1 " " 1,215 |
| S. Carolina, | 335,314 | 137 | 1 " " 2,447 |
| Georgia, | 283,697 | 134 | 1 " " 2,117 |
| Alabama, | 255,571 | 125 | 1 " " 2,045 |
| Mississippi, | 196,577 | 82 | 1 " " 2,397 |
| Louisiana, | 193,954 | 45 | 1 " " 4,310 |
| Tennessee, | 188,583 | 152 | 1 " " 1,241 |
| Kentucky, | 189,575 | 180 | 1 " " 1,053 |
| Missouri, | 59,814 | 68 | 1 " " 879 |
| Arkansas, | 20,400 | 21 | 1 " " 970 |
| Florida, | 26,534 | 12 | 1 " " 2,221 |
| Dist. Columbia, | 13,055 | 7 | 1 " " 1,865 |
| Totals, | 2,701,591 | 1,734 | |

Making 1 to every 1,557 in the Slave States.

By the above table we learn from the most reliable source of information at our command, (although allowance must be made for much error,) the number of insane and idiotic among the negroes of each state, and the ratio to the same, as well as arrive at the general results as before stated. By observing the results in the different states, it will be seen that the proportion of the insane quite regularly increases from the extreme south, where, in Louisiana, there is but 1 insane or idiotic in 4310; to the extreme north, where, in Maine, New Hampshire and Vermont, there is over 1 to every 50. The influence of climate by this would be indicated as considerable.

By a reference to the intellectual condition of the white population of the United States, as shown by the number of persons in each over the age of twenty-one years that cannot read and write, in the following table, also made out from the report of the census of 1840, it will be seen that where there is the least ignorance there is most disease of the mind. This does not hold true in every instance, but it is as a general rule the result.

TABLE No. 3,

Showing the white population of each State in the United States, the number of insane and idiotic, and the proportion they bear to the whole, the number of persons over 21 years of age who cannot read or write, and the proportion they bear to the whole.

| White Population. WHOLE NO. | | Insane and Idiotic. | Proportion to the whole. | No. white per. over 21 yrs. of age, that cannot r'd & write | Proportion to the whole population. |
|---|---|---|---|---|---|
| Maine, | 500,438 | 537 | 1 to ev. 932 | 3,241 | 1 " " 154 |
| New Hamp. | 284,036 | 486 | 1 " " 567 | 942 | 1 " " 301 |
| Massachusetts, | 729,010 | 1,071 | 1 " " 671 | 4,448 | 1 " " 164 |
| R. Island, | 105,587 | 203 | 1 " " 520 | 1,614 | 1 " " 65 |
| Connecticut, | 301,856 | 498 | 1 " " 606 | 526 | 1 " " 560 |
| Vermont, | 291,218 | 398 | 1 " " 731 | 2,270 | 1 " " 128 |
| New York, | 2,380,890 | 2,146 | 1 " " 1,109 | 44,552 | 1 " " 53 |
| New Jersey, | 351,588 | 369 | 1 " " 952 | 6,385 | 1 " " 55 |
| Pennsylvania, | 1,676,115 | 1,946 | 1 " " 861 | 33,941 | 1 " " 49 |
| Delaware, | 58,561 | 52 | 1 " " 1,126 | 4,832 | 1 " " 10 |
| Maryland, | 317,717 | 387 | 1 " " 823 | 11,605 | 1 " " 13 |
| Virginia, | 740,968 | 1,052 | 1 " " 704 | 58,732 | 1 " " 12 |
| N. Carolina, | 484,870 | 580 | 1 " " 836 | 56,609 | 1 " " 8 |
| S. Carolina, | 259,084 | 376 | 1 " " 689 | 20,615 | 1 " " 12 |
| Georgia, | 407,695 | 294 | 1 " " 1,386 | 30,717 | 1 " " 13 |
| Alabama, | 335,185 | 232 | 1 " " 1,445 | 22,592 | 1 " " 15 |
| Mississippi, | 179,074 | 116 | 1 " " 1,543 | 8,354 | 1 " " 21 |
| Louisiana, | 158,457 | 55 | 1 " " 2,881 | 4,861 | 1 " " 32 |
| Tennessee, | 640,627 | 699 | 1 " " 916 | 58,521 | 1 " " 11 |
| Kentucky, | 590,253 | 795 | 1 " " 742 | 40,018 | 1 " " 15 |
| Ohio, | 1,502,122 | 1,195 | 1 " " 1,257 | 35,394 | 1 " " 42 |
| Indiana, | 678,698 | 487 | 1 " " 1,392 | 38,100 | 1 " " 18 |
| Illinois, | 472,254 | 213 | 1 " " 2,217 | 27,502 | 1 " " 18 |
| Missouri, | 323,888 | 202 | 1 " " 1,603 | 19,457 | 1 " " 16 |
| Arkansas, | 77,174 | 45 | 1 " " 1,715 | 6,567 | 1 " " 11 |
| Michigan, | 211,560 | 39 | 1 " " 5,424 | 2,173 | 1 " " 90 |
| Florida, | 27,943 | 10 | 1 " " 2,794 | 1,303 | 1 " " 21 |
| Wiskonsin, | 30,749 | 8 | 1 " " 3,843 | 1,701 | 1 " " 18 |
| Iowa, | 42,924 | 7 | 1 " " 6,132 | 1,118 | 1 " " 38 |
| Dist. Columbia, | 30,657 | 14 | 1 " " 2,189 | 1,033 | 1 " " 29 |
| Totals, | 14,191,199 | 14,512 | mak. 1 to ev. 984 | 549,723 | mak. 1 to ev. 25 |

*Political and Religious* strifes and contentions, have to a much greater extent than all other causes, produced great and general excitement. On the principle already laid down, this fact would give them a prominent place among the causes of mental derangement. Not only do strifes in politics and religion exert an influence for weal or woe to the mind, but the form of government, as it gives tone to the feelings and caste to the sentiments of a people, may be considered as exerting an influence. Other things being equal, we would expect more insanity in a republic, than a monarchy, as the mind is here called into greater activity. Although few cases are found reported in this country as being caused by political influences, the great anxiety and inordinate excitements consequent upon them, undoubtedly here as in France, and other countries, do produce insanity. The reports of all institutions for the cure of the insane abound in cases produced by religious influences.

*War*, with its attendant train of evils and sorrows, is a fruitful source of insanity. During the troubles of France, this was particularly demonstrated.

*Civil State.*—That celebacy is favorable to the production of insanity admits not of a doubt, since at the ages when insanity is the most common, a majority of the good citizens of our country are married, and the reports of all our asylums and hospitals for the insane, show a greater number of single than married persons insane.

*Fright.*—Many remarkable cases of insanity by fright might be quoted. The effect is often instantaneous.

*Anger.*—The wife of an acquaintance of mine had been for years in the habit of giving unrestrained latitude to her anger, from trivial circumstances, flying into a violent rage at her husband. They had several times packed up their goods and prepared for a final separation, but the preparation occupied so much time and diverted her mind so as to allow her anger to cool and they would agree to remain together. Her fits of anger became more and more violent until she became entirely deranged. She forsook her home and husband, and wandered over the neighborhood from house to house, uttering the most bitter denunciations against him.

*Excessive study.*—By an inordinate application of the mind the brain is taxed above what the physical energies will support, and frequently an irritation is established in the brain which results in insanity.

I have already dwelt more fully on the causes of insanity than the limits of my article seem to justify, because in doing so it has been necessary to adduce many facts that will be found interesting independent of this connection. The other causes referred to are quite common, but from what has been said the reader will readily see the reasons why they should have this effect and I leave them.

FORMS OF INSANITY.

Under this head authors have generally made quite a multiplicity of distinctions, many of which serve much better to display the learning of the author, than to elucidate the subject. The different forms of the disease so often pass into each other without any manifest influence determining the change, that it is exceedingly difficult to make any very accurate distinctions, that will give a clue to the pathology of the case. However, as the peculiarities of these different forms of disease, may be important in directing the moral treatment, I will notice a few of the most prominent. They are *mania*, *monomania*, *melancholia*, and *dementia*. There are distinctions depending upon the organ, or part of the brain, or nervous system affected, not generally treated as varieties of insanity, which are much more properly entitled to the distinction, as derangements of the senses, of the intellect, and of the moral sentiments. That different parts of the brain as well as of the general nervous system, perform different functions, is beginning to be quite generally admitted. And if it be true, why not look to different parts of the brain for disease, when certain feelings, sentiments or faculties of the mind are deranged in their action?

*Mania.*—This form of insanity is readily recognized. It is characterized by a peculiar wild expression of countenance manifesting great anxiety, perturbation and excitement of the feelings, and is attended with a general derangement of the intellectual faculties. The eyes are injected with blood, and glare wildly. The features become more rigid and are subject to frequent contortions. The hair often becomes dry and stiff, and adds to the haggard appearance.

This form of insanity is the most violent and uncontrolable, but not by any means the most incurable. It often continues for several days, or weeks, or even months, and passes into one of the other forms of the disease. When this form occurs, it is generally at the onset of the attack. It is that form of insanity most generally attended by, and consequent upon excitement. Of the various forms of this chronic disease, mania is the most active.

Climate, and the peculiarities of the inhabitants have much to do in determining the character insanity assumes. Dr. Ray observes, "I have little doubt that in Great Britain and France, insanity assumes very much less frequently than with us, the form of intense and completely uncontrolable excitement, and when this condition does occur, it is of much shorter duration." Energy is one of the prominent characteristics of the American. He does everything he undertakes with all his might. He distinctly acts on the high pressure system; being accustomed to freedom of thought, speech, and action, constantly pressing forward with the brightest hopes to the highest destinies, while in health, it is but reasonable that we should find him when insane, the subject of the most ungovernable excitement.

*Monomania.*—The mind may be but partially deranged, that is in reference to but one or two subjects, while the other powers remain unimpaired. Although recognized as a distinct form of the disease by authors, it in fact differs from that of mania only in extent. It often precedes mania, and constitutes a premonitory symptom of its onset. Many persons are to the full extent of the meaning of the term, monomaniacs, who are yet capable of transacting business, and fulfilling competently most of the relations and duties of life. In fact many of the most distinguished persons have been partially deranged. Luther fancied that the devil was in him, and that he heard him speak. Butler in his celebrated Hudibras, refers to this in the following lines:

> "Did not the Devil appear to Martin
> Luther, in Germany, for certain."

Not only does slight disease of the brain frequently exist without destroying the mind, but it frequently has the influence of exalting its powers. Dr. Brigham says "In the writings of Fielding, Metastasio, Pope, Dryden, Rousseau, Madam Roland, Dr. Johnson, Byron, and many others, are descriptions of incipient madness, evidently drawn from their own sensations. Metastasio says, "when I apply with attention, the nérves of my sensorium are put in a violent tumult, and I grow as red as a drunkard." Pascal often sprang from his chair while composing his most celebrated works, seeing a fiery gulf opening by his side. Descartes was often followed by an invisible person, calling on him to pursue the search of truth. Benvenuto Cellini, saw a resplendent light hovering over his own shadow, and Raphael says, alluding to his celebrated picture—the transfiguration—that when engaged upon it he might be looked upon as an enthusiastic madman: that he forgot himself, and fancied he saw the whole action passing before his eyes. Cowper was decidedly insane, even at the time he wrote his most celebrated poems. All this time and for many years, he doubted the identity of his most intimate friend, the Rev. Mr. Newton. Cruden, the author of the concordance of the bible, was insane more than thirty years, during which time he prepared and published that learned and valuable work. Robert Hall might be mentioned, if not as an instance of the improvement of the mental powers by insanity, certainly as one in whom this disease did not injure them.

Numerous cases are on record in which great difficulty has been experienced by physicians and jurists in establishing insanity; so well and correctly did the subjects of it think and talk on almost all subjects, while in reference to one or two topics they were quite deranged.

Dr. M——, an incurable inmate of the N. Y. State Lunatic Asylum, is quite an intelligent correspondent of the Utica papers. He converses freely and sensibly on most subjects. The following "prayer for the insane poor," he wrote and presented me:

"O God, who declarest thy power! in commiseration mercifully regard me and restore me to saneness and composure amidst all my perplexities. To usefulness, respectability, individual and social happiness. To an ability to acquire and control the means for my respectable and honorable maintainance. To the rational enjoyment of the privileges, and to the faithful performances of the duties of an American citizen."

I heard him declaim "The Soldier's Dream" in a manner that would have done credit to to the soundest mind and the best student of elocution. In fact, no one, from all I saw, would have suspected him of insanity.

Almost every hospital for the insane has its preachers, its politicians, its sages who manifest their wisdom by silence, its philosophers and its poets. One of the best productions from one of the latter class, and they are numerous, is the following from a patient in the "Retreat," near York, in England. He, it seems, fancied himself destitute of heart, liver, brain, and everything else.

> "A miracle, my friends come view!
> A man, (admit his own words true)
> Who lives without a soul;
> No liver, lungs, nor heart has he;
> Yet sometimes can as cheerful be
> As if he had the whole.
>
> His head, (take his own words along)
> Now hard as iron, yet ere long
> Is soft as any jelly;
> All burnt his sinews and his lungs:
> Of his complaints, not fifty tongues
> Could find enough to tell ye.
>
> Yet he who paints his likeness here,
> Has just as much himself to fear,
> He's wrong from top to toe;
> Ah friends, pray help us if you can,
> And make us each again a man,
> That we from hence may go."

From what has already been said, it is plain that no regular distinctive symptoms can be given of monomania. It is any form of insanity partial in its extent.

*Melancholia.*—Dr. Elliotson says "there is no real difference between "mania" and "melancholia."

The distinction here tried to be made is to form a class of those cases in which great depression of spirits prevail. But no more appropriate is this, than to make a class for the suicidal, homicidal, loquacious, taciturn, the merry or the voracious; for almost all forms of insanity are liable at times to assume this character.

*Dementia* is that form of the disease that destroys the mind, or so far affects the brain as to suspend the mental manifestations. It is most common in old cases, where from long continued disease the brain becomes more and more under its influence, until there is a complete want of mind. Although it occasionally is found in the early stage of the disease, most authors deny the existence of acute dementia. When it does occur, I apprehend it is from a prevalence of disease through the whole brain, to the extent of entirely suspending its appropriate action as organ of the mind. I saw a case last spring in the Pennsylvania Hospital for the Insane, that I supposed to be of this character.

There is little hope of cure in long cases of dementia, especially in those that have gradually assumed this character.

There is another form of insanity that has recently attracted much attention. It is of the utmost importance, not only in a pathological and therapeutical, but also in a medico-legal point of view. It is called *moral insanity*, and is a derangement of the moral sentiments and feelings without an overthrow of the reasoning or perceptive faculties.

Great destructiveness, acquisitiveness, self-esteem, love of approbation, sexual desire, &c., are often found where they may not be said to constitute insanity. But when these propensities become so strong as to entirely control the action of the individual, in spite of his better judgment, (and they often do) while all his moral powers are brought unavailingly to bear against them, he is certainly insane. When an individual feels an irresistable impulse to do any unlawful act contrary to his better judgment, he is deranged; but the case becomes much more manifest when the act runs counter to the known and established laws and feelings of his own nature: as when a parent is impelled to the destruction of his own child, &c.

Such cases require in the jurist and medical witness great care in investigation, and sound and enlightened judgment in decision, lest on the one hand they condemn the irresponsible and consequently innocent, or on the other they let the guilty go free.

Hereditary conformation, education, and the power of habit have much to do in predisposing to this form of insanity.

Numerous instances are on record where the excessive indulgence resulting from hereditary disposition, or vicious education, of one or more of the propensities of our nature, have so strengthened them as that they have acquired the complete control of the whole man; or in other words produced moral insanity.

I believe the day is not far distant when an investigation of the pathology of insanity will give us a more rational classification of its different forms, founded upon the disease of the brain and the parts affected, instead of the peculiarities of the mental manifestations of that disease.

### SYMPTOMS.

When an individual, not laboring under fever of a grade to produce delirium—sees objects that have no real existence—when a marked change comes over his feelings, his desires, his sentiments and his disposition,—when in his attempts to reason, that which was formerly clear and explicit to his mind, becomes confusion and error—when from plain facts he draws wild and erroneous conclusions—when he fancies himself or the objects around him to be different from what they are—when he supposes he has converse with unseen spirits, with the inhabitants of the other world or with the dead—or when he fancies himself an inhabitant of a far distant country, of the moon, or of eternity—although the wreck of mind may not be established, it is time for him and his friends to suspect that he is insane; and often by a timely resort to the rules of treatment hereafter laid down, the dread calamity may be averted.

It is important to detect the earliest symptoms of insanity, for it is in this stage that our remedies are the most potent and the cure most speedy. Often man may prevent insanity by understanding the tendency of his mind to the disease. And even where it is established to some extent, he often has the power to control it. The remarkable case of Nicolai of Berlin, who saw for months together vast numbers of human forms around him, and yet so far controlled his mind as to remain entirely sane, is a case strictly in point.

It cannot be expected that unaided, the patient can control his insanity where the derangement is general, yet here as we shall hereafter see, much controlling influence may be exerted by moral treatment.

Insanity sometimes begins suddenly. This generally occurs in puerperal cases in which it mostly assumes a violent form, corresponding with the activity of the disease that produces or constitutes it. In other cases it comes on gradually, being first manifested by oddity of manners, strange opinions, or absurd fancies. These frequently are but an exaggeration of notions previously entertained.

A steam doctor in Ohio, of some notoriety, who had long indulged the most illiberal opinions in reference to the regular practice of medicine, after laboring under an affection of the lungs until much reduced, consulted a physician of eminence in the town where he resided. Soon after he supposed himself poisoned. He labored under constant apprehensions lest some of the physicians should slip some of their poisonous drugs into his food or drinks, although no one of the faculty went near him. He was removed to the Ohio Lunatic Asylum, where it was with great difficulty that Dr. Awl could so far gain his confidence as that he was willing to submit to the course of treatment by which, soon after, his mind was restored.

*Physical signs.*—As has been seen, there are various forms of disease attendant upon insanity, either acting as causes or consequent upon it. Of the first, sufficient has been said under the proper head. The latter we find to be various, and by no means constant.

In some cases there will be a frequent, and in others a slow pulse, while sometimes there seems to be but little disturbance of the circulation. As a general rule the pulse is not to be relied upon as affording any index to the pathology or treatment of the disease. Dr. Cox says, "whenever it exhibits very considerable changes without any obvious causes or corresponding symptoms, sudden death frequently closes the scene."

When insanity occurs in connexion with other diseases, they frequently modify the circulation, when its changes are pathognomonic of the attendant disease, and not of the insanity. Dr. Rush placed great stress upon the pulse in mental derangement. His theory of the disease was, that it depended upon vascular excitement; the practice deduced from which, was copious bleeding—an error in theory that to some extent modifies most prejudicially the treatment of the disease in private practice to the present day.

The head is frequently too hot—the face flushed and the eyes injected. It is sometimes the case, that while the pulse at the wrist is feeble, the carotids throb forcibly—denoting an unnatural determination of blood to the head.

The secretions are often perverted, diminished or increased. The tongue is generally coated and the saliva changed. The appetite is exceedingly variable. Sometimes the inordinate voracity of the patient is the first index of disease. In other cases there is not only a loss of appetite, but a great aversion of both food and drink. In many cases the only means of introducing food enough into the stomach to prevent death from starvation, is that adopted in several of the hospitals. The food is prepared in a liquid form—the tube of the stomach pump passed down the throat, and by means of the pump the food is forced down.

The external senses are often diseased—vision is impaired, perverted, or the eyes become extremely sensitive to the influence of light. The ears are affected, and the patient hears strange sounds—a roaring sensation in the ears, persons talking, &c. The taste is often perverted, and the olfactories suffer from disagreeable odors. The sense of feeling, too, undergoes various changes.

In some cases there is much stupor, but more, generally, there is much restlessness; and in such, frequently, the loss of sleep hastens on the disease. Patients often go days and even weeks, without sleep; the disease of the brain acting as a substitute for that restoration of energy brought to the weary by "balmy sleep."

PATHOLOGY.

It will not be necessary to enter into an argument to prove that the brain is the organ through which the mind manifests itself—the instrument through which its operations are performed. This is admitted. Nor will it be necessary after this admission, to adduce arguments to prove that a diseased condition of the brain will give rise to imperfect and deranged actions of the mind, any more than it would to prove that an injury of the eye would impair vision.

Then, if disease of the brain will derange the mind, we are authorized to look for it whenever the mind is deranged. The disease in insanity, as has already been said, is physical, and any other supposition would lead to the position that the mind is subject to decay, and would rob it of its noblest attribute, immortality.

When the functions of any organ are deranged, we look to the organ itself for the cause; when hearing is impaired we refer to the ear, and when sight is interrupted we look to the eye. Whether the disease be primarily in the organ itself, or is produced secondarily by morbid actions in other parts of the system, we refer to the organ whose functions are deranged for the proximate cause. When a secretion is arrested, perverted, or becomes too profuse, we say a morbid condition of the gland by which it is secreted is the cause. And so with reference to the manifestations of mind; when we find them deranged we infer a morbid condition of the brain. *All mental derangements are but symptoms of disease in the brain*, which may be either primarily affected or diseased by the derangement of some other part of the system with which it sympathises or is in close dependence. By a reference to the part the brain acts in the animal economy—being the sensorium commune,—we see how close are its dependencies, and how extensive its sympathies. From it emanates the nervous influence which gives vitality to all parts of the system. To it flows through the innumerable bonds of connection formed by the nervous system every impression received, and consequently every morbid action in the system must have a greater or less influence over it, while every morbid impression upon it must exert an influence over the whole system. Thus, when the stomach fails to perform its function, nutrition is suspended, and the necessary support is withheld from the brain in common with other parts. That supply of healthy blood so necessary to its normal action, and which is one of its natural stimulants is not furnished, and imperfect action is the consequence. Again, the sympathy between the brain and stomach, through the pneumogastric or par-vagum nerve, is most important. How speedily an injury of the brain affects the stomach, and how certainly disease of the stomach affects the brain. With the heart, the lungs, and in fact with all parts of the system, these relations are more or less closely sustained. But it is only when the brain itself suffers a lesion, that we have insanity. It may be considered a disease of the nervous system; and very properly has Dr. Good placed it at the head of his class neurotica.

As the structural changes that takes place in the brain and its membranes in many cases, are by no means constant or regular, we are led to conclude that insanity is produced most generally by irritation; a term, which some authors of eminence, make synonymous with inflammation, in which definition I do not concur. That inflammation, properly so called, will derange the mind, when seated in the brain, is a fact that no one will deny; for the delirium produced by phrenitis and arachnitis are positive evidences of it. The form of mental derangement generally understood by the term insanity, is slow in its action, in many cases entirely free from producing any inflammatory excitement in the system, and is often attended by great depression of the energies and action of the vascular system. Its periodical form, too, would do much towards establishing irritation as the peculiar form of morbid action. The character of many of the causes may also have an influence in directing our investigations of the pathology of insanity. When we remember that many of them act gradually in producing the morbid condition—that the inception of the disease is attended in many instances simply by functional derangements, and that many patients labor under the disease for twenty and thirty years and even longer, whose brain after death presents in almost all respects a healthy appearance, we can but conclude that this disease is one of irritation.

The manner in which the disease is produced, varies much in different cases, and from different causes. Sometimes the irritation is reflected, as when the close student, by too great exertion of the brain, robs his stomach of the nervous energy necessary for healthy digestion, becomes dyspeptic, by continuing the mental application involves the brain still more and more in disease, the disordered stomach adds its influence in increasing the morbid action. He first becomes melancholic and finally insane. Dyspeptics are generally hypochondriac, and often labor under a condition of the brain that closely approximates insanity. The insane very often labor under derangements of the stomach and bowels, and many of them are dyspeptic. Not only does the dyspepsia often aid in the production of insanity, but disease of the brain produces dyspepsia. Many other diseases have this kind of reciprocal action with insanity, which in its treatment should always be borne in mind.

That different parts of the nervous system perform different functions, is established beyond a doubt. There is one class appropriated to the function of muscular motion, part of which only, is under the influence of the will. Another class endowed with sensation, which is subdivided into as many parts as there are external senses, and each of these nerves perform their appropriate function only. They originate from separate portions of the brain, which seems to indicate that it is a plurality of organs. We also find from various observations made upon the brain in health and disease, that its different parts perform different offices. That although a portion of one hemisphere may be removed without impairing any of the faculties of the mind, no case is on record where corresponding portions of the hemispheres, have been diseased, without destroying or deranging one or more of those faculties. From these facts we are justifiable in the conclusion, that different portions of the brain being diseased, will produce different forms of insanity. Although cases occur in which all the faculties of the mind are equally deranged, this is by no means a general circumstance. Mostly some one, two or three of the faculties are particularly affected, while the rest seem to be but little disturbed. When this is the case we infer that the part of the brain appropriated to the use of those faculties of the mind, is the seat of disease. And this is corroborated by observations on the treatment of insanity. When on a particular subject a patient is insane, the best moral treatment is to divert the mind from that subject, and allow the part of the brain through which it acts, repose. If the position taken was not true, any form of mental action would be injurious while the brain was diseased, but experience abundantly establishes the propriety of mental employment, provided, it be directed out of the channel of the disease.

That a pathology of insanity founded upon the parts of the brain affected, will ultimately be permanently established, I have no doubt. And when it is, both the moral treatment of the disease, and the prophylactic means used, will be to a great extent governed by it.

*Post Mortem dissections* occasionally discover but little evidence of disease in insanity, from which some have been ready to doubt the seat of disease being in the brain. But are not dyspepsia and asthma located in the stomach and the lungs? And how often is it the case that the morbid appearances of either of these organs after death from these diseases, give no evidence of their having existed. However, when insanity has continued long, there is evidence of disease generally in the brain and its membranes.

In many cases of insanity, connected with paralysis, there are signs of inflammation in the brain.

In recent cases there is often no morbid appearance after death, but changes in the consistence and appearance of both the cortical and medullary matter of the brain, and thickening and adhesions of its membranes are common in those of long standing.

The heart and lungs are frequently diseased. Hypertrophy of the heart, and phthisical degenerations of the lungs, are said to be quite common.

Gastro-enteritis has been found to have existed in many cases. But in reference to all these, we should expect great derangements in almost all parts of the body, from the diseased influence the brain in insanity must exert on the system. And no doubt many cases die from insanity by the establishment of disease in different parts of the system, through this morbid influence.

### CURABILITY OF INSANITY.

The reports of the different hospitals and asylums for the treatment of insanity, abundantly establish the curability of the disease. From seventy-five to ninety per cent, of the recent cases admitted are cured by most of them. And their physicians all concur in the opinion, that when taken in its early stages, it is as curable as a fever or pleurisy.

Although many disadvantages attend its treatment in private practice, a judicious resort to the means within our control, will enable us to cure quite a majority of the recent cases, even here.

### TREATMENT OF INSANITY.

I propose to confine my remarks under this head principally to what will be appropriate in private practice. It is properly divided into the *moral* and the *physical* treatment.

*Moral Treatment.*—As soon as symptoms of insanity are discovered, the patient should be placed under treatment, which in many respects must be varied to suit the peculiarities of the case. But he should always as far as is practicable, be removed from under the influence of the exciting cause. When in its incipient stage, the proper remedies to correct the physical derangements present, and a removal from the influence of the exciting cause, will often prevent the full development of the disease.

If the cause be a moral one, any means of directing the mind into another channel of thought, will be appropriate as a preventative, or a means of averting the full development of disease.

The most effectual means of doing this will be a change of pursuit, removal from home, traveling, &c. Even when the disease is fully established this will be necessary, and should be attended to without delay. It may be laid down as one of the first and most important points in the moral treatment of insanity, to remove the patient from amongst his friends, and particularly from his own family if he have one. This is necessary for various reasons. *First*, that the associations that have been formed in the development of the disease, which may have had an influence in producing it, may be broken up. *Secondly*, because these associations, if not acting as a cause, have become so intimately connected with the diseased train of thought, that they constitute a part of it. *Thirdly*, because in a change of scene new objects present to the mind and institute new channels of thought, as well as tend to interest the mind. *Fourthly*, because strangers have much more influence in governing the insane, than those with whom they have been accustomed to associate on terms of intimacy and equality. They often are highly incensed at authority exercised over them by those from whom they have been accustomed to kindness, which from strangers they would receive kindly; and this is one of the reasons why the insane so universally regard those who have previously been their best friends, in the light of enemies.

*Kindness.*—It is of the utmost importance that the insane be treated by all, with the utmost kindness. In selecting an attendant to take charge of a patient, we should be particularly careful to obtain one who can sufficiently appreciate the condition of his patient, not to become irritated at his words or conduct, however insolent they may be, who would understand and appreciate the fact, that *the laws of humanity and of kindness are the only rules that should be applied for the government of the insane.* However much the reason may be dethroned, there are few, very few, who cannot be made to feel those heaven born influences that flow from the benevolent heart. He should be one, too, who while he would take care for the physical comforts of his charge, could also direct his mind from those "carking cares," wild fancies and hallucinations, that else would form his theme of converse and of thought by day and by night.

*Employment.*—Let something be devised to keep the patient employed; and if he is able, and has been used to labor with his hands, this will be the best means. While the hands are employed busily the attention will be directed to the work; and although much effort may be necessary to effect an application to it, most persons can eventually be interested, which will abundantly reward for the pains by engaging the attention and invigorating the system.

The cultivation of a garden, labor in the field, or some mechanical occupation for the male—sewing, knitting, spinning, washing, ironing, scrubbing, and the like domestic labors for the female, will be entirely appropriate, where the ability and inclination will allow. No harsh measures should be used to induce the insane to work, or for any other purpose.

When ability or inclination show the impropriety of this kind of employment, many amusements may be resorted to, and even with those who labor they should be introduced for recreation.

The insane are often fond of reading, which is both an interesting and useful amusement. They often take great interest in histories, works of fiction, and in reading newspapers. Where there is an inclination of this kind it should always be encouraged; however, with a care not to place any work in their hands that treats upon any of the subjects upon which they are deranged. In fact, in selecting employment and amusements for them, care should be had in every case to see that they divert the mind from those subjects upon which they generally dwell.

Schools in hospitals are becoming quite popular as a means of entertainment and instruction. I saw a most interesting exhibition at the N. Y. State Lunatic Asylum last spring, in which the insane scholars deported themselves with great propriety, read many pieces of their own composition, recited lessons and gave some fine specimens of declamation. Dr. Brigham assured me, that many of them improved rapidly. Some philanthropic individuals in Europe are doing wonders in developing the minds of idiots by educating them. It is said they succeed in teaching many, rules of behavior, who appeared previously almost entirely destitute of mind.

By giving the insane lessons, they are entertained, and the mind kept steady, which favors recovery. Geography, history, language, reading and writing, form good studies for the insane. Drawing and painting too, are interesting to those who have a taste for them.

An intelligent lady, a few years ago, having long been a professed christian, was so rejoiced at the conversion of her husband, who had been an avowed infidel, that she went all lengths with him in devotions and leading a life of self-denial. The subject of religion was their constant theme. They had family prayer three times a day, and fasted twice a week; while she at the same time was suckling a young child. Her health began to decline, she complained of general debility. A loss of appetite and a torpid condition of the liver soon followed. For a time her devotions became more fervent, her religious enjoyment scarcely knew any bounds, and the whole religious community rejoiced in her spiritual prosperity.

But her physical system was too much worn down by fasting, suckling and application, to bear the mental excitement with impunity. She lost the evidence of her acceptance with God, and her enjoyment was changed to despair. Her countenance manifested it in every feature; and her supplications for mercy, by their fervency and earnestness, corroborated the expression. Her sinfulness in having professed a change of heart, when (as she said) she had not, and all the fancies and real errors of her life were brought up in fearful array before those she addressed. She would earnestly solicit advice of her friends, and often inquire whether they thought it possible for her to receive pardon. Her husband reasoned with and prayed for her, and her pious friends and neighbours held prayer meetings at the house for her especial benefit; but little thinking, that, in calling for Divine aid, they were violating one of the established laws of nature, which man may not disregard with impunity. Of course, under this she became worse and worse. She even thought of self-destruction, and had made preparations for the awful act.

When I took charge of the case, it was with the utmost difficulty that I could for a moment divert her mind from the subject of religion, and from brooding over her deplorable condition,

I removed her from home, forbade conversation on the subject of her condition or religion in her presence, used the physical remedies indicated by the derangements of the general system, gave liberal doses of morphine at night to secure repose, for she, as is usual in incipient insanity, passed sleepless nights. I set her to work with her pencil, which she plied with much taste and skill, part of the day, and required her to recite a lesson in French in the evening. These occupied much of her time, and she soon became interested in them. She rapidly improved, her countenance brightened up, she slept soundly, and in the course of a few weeks was quite comfortable. It was some time, however, before the subject of religion could be introduced in her presence without causing much agitation of mind. And it was months before she could attend religious service without great excitement. However, by carefully avoiding the subject, she in time became entirely healthy, and able to converse on all subjects with intelligence and interest.

*Restraints.*—The too common practice of confining patients in block houses, jails and tight rooms, or of confining them in chains, where they are left alone not only hour after hour, but day after day, and month after month, without more privilege of seeing their fellow creatures than is necessarily afforded by those who supply them with food, or visit them out of idle curiosity to see a "crazy person," is not only calculated to aggravate and confirm the disease, but is most cruel and shocking to humanity. All cases, having the ability to bear the expense, should have an attendant immediately employed, and those unable should be furnished by the county in which they reside, who could entertain, console, and by acts of kindness, calm his troubled mind, using as little restraint as it is possible to get along with.

An appliance for the violent, that renders them perfectly harmless, yet allows them freedom in walking about, is the hand-muff or camisole. It is made of leather in the form and shape of a section six inches long of a sleeve, with a strap at each end, passed round the margin through slits cut for it through the leather. The hands are both passed into this, and the straps are drawn sufficiently tight around each wrist to prevent the hand from slipping out, and confined by a little padlock or any other contrivance the ingenuity of the attendant may invent. Another mode of confining the hand that answers equally as well in most cases, is prepared by making a frock or coat of strong linen, with sleeves about twice the length of the arm. The hands in the sleeves are then crossed over the breast loosely, and the ends carried around and tied on the back. With such confinement as this and proper attention, no one need fear the most violent; and I repeat, it is cruel to use more restraint than is necessary, for safety, and protection and comfort of the patient.

PHYSICAL TREATMENT.

Under this head I do not propose laying down rules of treatment for the various complications of disease with which insanity is often attended. The general principles of treatment, applicable to each particular case, must be our guide.

Although many authors seem to regard medical treatment lightly as applied to the cure of insanity, there are many remedies of great utility as we shall hereafter endeavor to prove. There is much less confidence in these remedies generally, in Europe than in this country, probably owing, in part, to the fact that insanity there generally assumes a different character from that in this country, as has already been pointed out.

The moral and physical treatment should go together; and the sooner after the attack they are applied the better the prospect of cure.

*General bleeding.*—There are few cases of insanity that are not made decidedly worse by the free abstraction of blood. It is a source of general regret in our American Institutions for the insane, that practitioners so generally resort to it. It may for a time check the excitement; but it generally, when reaction takes place, returns with unabated violence, and with the disadvantage of having the irritability of the system much exalted, while the energies are depressed. If the disease were, as some suppose, an inflammation of the brain, or, as Rush and his followers contend, dependent upon vascular excitement, this would be one of our most valuable remedies. But experience, "the touch stone," is loud in its condemnation of bleeding as a general practice. In many cases thus treated, fatal depression of all the energies of the system is the consequence. While under the excitement of insanity, the system will bear copious bleeding, and may be reduced so low before the practitioner is aware of it, that when the excitement, which fills the whole system with artificial energy, is subdued, in spite of tonics and stimulants the patient sinks to rise no more.

That there are cases in which general bleeding would be proper on account of complications, as when connected with inflammation, is certainly true; but great care must be had not to mistake the excitement simply consequent upon insanity, for a symptom of inflammation.

*Local bleeding*, by cupping and leeching, is a valuable means of diminishing the determination of blood to the head, and of reducing the action there, but must be regarded as a means of secondary importance.

*Cold applications to the head.*—These either in the form of the douche, or of ice, are valuable remedies. Many cases have been reported as cured by pouring cold water on the head alone; but it is when taken in connection with other remedies, and during excitement, that it is most valuable.

The douche may be given conveniently by filling a coffee-pot with cold water, and allowing the stream poured out of the spout to fall some distance on the head. This frequently calms the excitement, checks the flow of blood to the head, and allows the patient a period of repose. By repeating as the excitement returns, in periodical cases, much influence may be exerted in keeping the patient calm, and of course in relieving the disease.

*Baths.*—Amongst the means of removing disease from the skin, and of diffusing the circulation so as to equalize it,—and many if not most cases require remedies of this description—bathing will be found one of the most efficient. While I shall not stop to discuss the truth or error of the doctrines of Prisnitz, I am willing to award to the bath great efficacy in the cure of diseases.

Warm and cold baths are used in all well regulated hospitals for the insane, and of course with decidedly beneficial effects, or they would long since have been discontinued.

Where there is coldness of the extremities, and determination to the head, the warm bath given, while cold is applied to the head, has a most salutary effect and should often be repeated. The cold bath in many cases acts as an excellent tonic, invigorating the system and should often be used.

*Blisters.*—These, may, in some cases, be valuable in two ways: by establishing a drain from the system in cases arising from the healing of issues or the sudden arrest of perspiration; and by acting as a counter irritant However, in most cases, there is danger of their increasing the irritability of the system and aggravating the disease.

*Setons and Issues*, may serve as counter-irritants, and are more appropriate in chronic cases than blisters. They are not in as high repute now as formerly.

*Emetics* are not in high repute in the treatment of insanity *per se*, but often are appropriate in the diseases that attend it, which should always receive prompt attention. Those producing the least prostration and general irritability, should always be preferred. Ipecacuanha is one of the best. And I have no doubt the sanguinaria canadensis, on account of its promptitude of action and invigorating influence on the stomach, would be found valuable.

*Nauseants* may serve to check the general excitement; but the distress they produce and the irritability attendant upon their use, are sufficient reasons in most cases for laying them aside.

*Cathartics.*—There are many cases of insanity attended with derangements of the bowels, in which this class of remedies is valuable. But in reference to the curative treatment of insanity itself, there is not much reliance to be placed in purging. In its effect, it is too closely allied to the other depletory measures to which we have referred, to be generally advantageous. Where there is constipation, much may be done towards remedying it, by attention to diet, and the use of gentle laxatives. Where there is a torpid condition of the liver, Blue Pills, Cook's Pills, and such preparations as the peculiarities of the case may indicate, should be resorted to. Dr. Woodward highly recommends the use of guaiacum, either in the form of tincture, or the powdered gum, which latter he prefers.

*Diaphoretics.*—The skin is very prone to unhealthy action in insanity; in the early stages, being often hot and dry; to remedy which, sudorifics are valuable.

There is no article of this class that has higher claims to our confidence, than the Dover's Powders, (or what is but a modification of it, a combination of morphine and ipecacuanha,) used with carbonate of ammonia. However, the various preparations used to promote action of the skin, should be suited to the particular indications of the case. Where there is much excitement, the preparations of antimony may be admissible; where there is debility and want of tone in the stomach, the chamomile flowers, (anthemis nobilis) boneset, (eupatorium) pleurisy root, (asclepias tuberosum) guaiacum and remedies of this class may be appropriately resorted to.

*Narcotics.*--Dr. Woodward says, "by far the most useful remedies in active mania after the system is prepared for their use are narcotics."

The pathology of the disease being, as I have shown, that of irritation, we would, *a priori*, infer that this was the most appropriate class of remedies for its cure; and experience confirms the doctrines in pathology, by showing the fitness and success of the treatment. Often large doses of these remedies are required before the system can be brought under their influence; and it is scarcely necessary to say, that unless they are used to this extent, not only the object of their use will not be attained, but injury will be done to the patient by their administration.

*Morphine* stands at the head of this class of remedies. It should be given in solution, and the dose regularly increased until it produces tranquility of the system, and secures repose. In those cases in which sleeplessness is one of the most troublesome symptoms, and they are numerous, morphine and other preparations of this class are invaluable. When the effects of the remedy are secured, they can generally be maintained without much trouble, by continuing the use of the medicine, and frequently in the course of a few weeks the dose may be much reduced, and finally suspended, on account of the complete recovery of the patient.

The two ends of calming the system, and promoting the action of the skin, may be attained frequently by the prescription given as a diaphoretic. But the combination should only be used while the skin demands it; as the anodyne should be continued, so that the patient shall be continually under its influence. The amount of morphine necessary to be used cannot be given. In some cases it will seem almost incredible to tell the quantity necessary to produce its effect. It is scarcely necessary to say that care must be had in the use of this remedy, not to give it when there are any strong counter-indicating symptoms.

*Datura Strammonium*, has had considerable reputation with some practitioners. In epileptic cases it is said to answer a valuable end, where the system is kept so far under its influence as that the pupils of the eyes are affected by it. It may be given in tincture of the seed or in the extract.

*Cicuta.*—This remedy enjoys some reputation, and is used in the hospitals in combination with different preparations of iron, it is said with great advantage, and especially in the older cases, and those attended with neuralgic affections. It must be used in large doses to do any good; for unless it produces the "*heavy dull* pain over the eyes," and vertigo, which characterizes its effects, it will be useless to give it.

*Hyosciamus.*—From some recent researches in reference to the effects of this drug, which go to prove that its influence over the circulation in the brain, is different and opposite to that of opium, the former diminishing the flow of blood to the head, we would expect it to be of great service in many cases of insanity, Combining the anodyne with the influence of diminishing the circulation in the brain, we should expect that in almost all cases where the preparations of opium are forbidden, this would be appropriate. Although not so potent as morphine, in many cases it will secure tranquility of the nervous system and sleep.

*Strychnia.*—This is the great remedy for paralysis, and as many cases of insanity are attended by it, we would infer its applicability. It is a powerful remedy, and must be used with care. The dose should be gradually increased from what is ordinarily given, until it produces its peculiar effect—a sense of constriction in the stomach.

*Camphor*, with opium, especially, has great influence in controling the venereal appetite, and will be found particularly applicable in cases attended with priapism, where there is an indulgence in "the secret vice," and in nymphomania.

*Tonics.*—These are valuable to restore the system in cases worn down by excitement, and where there is a debilitated state of the digestive organs. The preparations of iron, bark, columbo, quassia, &c., in combination with aromatics, in the form of tincture, are amongst the most valuable.

*Quinine.*—This great febrifuge, the effects of which are now undergoing an investigation, can scarcely be passed over in the treatment of any disease in the great valley of the west. Our diseases are so often modified by the prevalent malarious influence of the country, for the cure of which quinine stands pre-eminent,—almost bearing the character of an entire specific—that it becomes us in the treatment of insanity, as in other diseases, to detect those symptoms arising from this influence and meet them. And whether quinine is a tonic or not, a stimulant or a sedative, it matters but little, if where we find traces of miasmatic disease it will remove them.

